# A qualitative study on the term CAM: is there a need to reinvent the wheel?

**DOI:** 10.1186/1472-6882-12-131

**Published:** 2012-08-21

**Authors:** Isabelle Gaboury, Karine Toupin April, Marja Verhoef

**Affiliations:** 1 Department of Community Health Sciences, University of Calgary, Calgary, Canada; 2 Department of Family Medicine, Université de Sherbrooke, Sherbrooke, Canada; 3 Centre de recherche clinique Étienne-Lebel, Centre Hospitalier Universitaire de Sherbrooke, Sherbrooke, Canada; 4 Department of Epidemiology and Community Medicine, University of Ottawa, Ottawa, Canada; 5 University of Ottawa, Institute of Population Health, Ottawa, Canada

**Keywords:** Definition, Term, Focus group, International experts, Integrative healthcare

## Abstract

**Background:**

As complementary and alternative medicine (CAM) has developed extensively, uncertainty about the appropriateness of the terms CAM and other CAM-related terms has grown both in the research and practice communities. Various terms and definitions have been proposed over the last three decades, highlighting how little agreement exits in the field. Contextual use of current terms and their respective definitions needs to be discussed and addressed.

**Methods:**

Relying upon the results of a large international Delphi survey on the adequacy of the term CAM, a focus group of 13 international experts in the field of CAM was held. A forum was also set up for 28 international experts to discuss and refine proposed definitions of both CAM and integrative healthcare (IHC) terms. Audio recordings of the meeting and forum discussion threads were analyzed using interpretive description.

**Results:**

Multiple terms to describe the therapies, products, and disciplines often referred to as CAM, were considered. Even though participants generally agreed there is a lack of optimal definitions for popular CAM-related umbrella terms and that all terms that have so far been introduced are to some extent problematic, CAM and IHC remained the most popular and accepted terms by far. The names of the specific disciplines were also deemed adequate in certain contexts. Focus group participants clarified the context in which those three terms are appropriate. Existing and emergent definitions of both CAM and integrative healthcare terms were discussed.

**Conclusions:**

CAM and other related terms could be used more effectively, provided they are used in the proper context. It appears difficult for the time being to reach a consensus on the definition of the term CAM due to the uncertainty of the positioning of CAM in the contemporary healthcare systems. While umbrella terms such as CAM and IHC are useful in the context of research, policy making and education, relevant stakeholders should limit the use of those terms.

## Background

Over the last 30 years, the fields of complementary and alternative medicine (CAM) research and practice have grown substantially, both at national as well as international levels. The growing acceptance of CAM by conventional practitioners and the increasing recognition that several of these therapies and disciplines play a prominent role in the health of patients have significantly impacted its use and study. However, public debates as well as the scientific literature suggest the need to revisit the terms and definitions used to refer to CAM practitioners, therapies, and products that have framed research and practice to date [1-6]. Facilitating meaningful change in healthcare terminology can be complex and challenging. The term CAM represents a particular challenge, in part because the topic is politically charged, but also because depending on the healthcare system context (national and international), its meaning changes.

### Plethora of terms and definitions

Since 1990, several definitions of CAM have been offered, particularly among healthcare researchers. Complementary medicine was originally defined as a “diagnosis, treatment and/or prevention that complements mainstream medicine by contributing to a common whole, by satisfying a demand not met by orthodoxy or by diversifying the conceptual frameworks of medicine” [7]. Then, the term *alternative* made its introduction and CAM was used to refer to “practices neither taught widely in US medical schools nor generally available in US hospitals” [8]. When the popularity of the field and use of the term of CAM increased, especially among the general public, and CAM disciplines such as chiropractic and osteopathy made their appearance in university curricula, the definition evolved into “*types of therapies or products that are currently not considered to be part of conventional medicine*” [9]. However, this is a description by omission: it describes CAM by what it is not.

Nevertheless, definitions of CAM have become inclusive in other ways. Often definitions acknowledge cultural context, since what is considered CAM in Western medicine might be considered mainstream in other countries (for instance, acupuncture in North America versus China). Zollman’s definition is a good example: “*Complementary and alternative medicine (CAM) is a broad domain of healing resources that encompasses all health systems, modalities, and practices and their accompanying theories and beliefs, other than those intrinsic to the politically dominant health system of a particular society or culture in a given historical period*.” [10]. Similar to the World Health Organization’s definition, these definitions portray CAM as a moving target, which represents a significant limitation in our contemporary multicultural societies where different healthcare approaches are available [11]. Moreover, culturally-specific definitions may preclude the research community from gathering all possible evidence on CAM and its safe and effective application in patient care [6]. As a consequence, this lack of consensus limits the healthcare practitioners’ ability to practice evidence-based medicine.

Wieland and colleagues have recently published a working definition of CAM for the Cochrane Collaboration, consisting of a closed list of therapies and products (e.g. the superseding topics: alternative medical systems, natural product-based therapies, energy therapies, manipulative and body-based methods, and mind-body interventions) that were deemed relevant to be classified as CAM [12]. As opposed to previously published theoretical definitions (on which most CAM definitions depend), this definition has the advantage of clearly identifying what should be considered CAM, thus creating an objective and reproducible operational definition for CAM. Nonetheless, as recognized by the authors, this definition is still subject to re-evaluation over time and its meaning depends on the dominant healthcare system; two stipulations that seem unavoidable when it comes to formulating a working and internationally-accepted definition. Interestingly, this working definition is similar to the one originally proposed by the NCCAM and recently revised by its representatives, showing how little agreement there exists on terminology in the research community. Ultimately, this list of therapies is probably as close to an operational definition as the research community has come.

In addition to the multiple definitions used to refer to these therapies, products and disciplines, the term CAM itself is puzzling. First, it combines two mutually exclusive terms: “complementary” suggests it can be used in tandem with biomedicine, while “alternative” means a substitution for biomedicine. Moreover, a specific therapy could be used both in combination with conventional care, or on its own as an alternative to conventional care. Second, many consider the term CAM inadequate because of its marginalizing implications. On the one hand, the definition borrows terminology from biomedicine and is comparable to it, but on the other hand, it is wholly different. The term CAM is often redefined to serve – or exclude – a specific group of individuals, therapies or products from biomedicine, and to carry different meanings for different people (e.g. chiropractic and osteopathy are sometimes, but not always, included under the term CAM) [13]. As a result, different communities (practitioners, educators, and the public) sometimes turn to terms other than CAM to alleviate these issues, such as holistic medicine, traditional medicine, functional medicine, and most recently, complementary and integrative medicine, to name but a few.

To address these concerns, some recommend that all therapies that aim at improving the health of individuals be included under the term *integrated* healthcare; a term that would eliminate the terminological barriers between conventional and unconventional care. However, the terms *integrative* healthcare (IHC) and *integration of care* have emerged in parallel, adding to the confusion since there is no consensus on their definitions. Moreover, these terms are commonly used for many other purposes, which are not always associated with CAM. For example, *integration of care*, although no shared definition exists, [14] is often used to refer to healthcare services that are centrally coordinated or combined with various types of conventional care [15].

### Consequences of plurality

Unfortunately, the plethora of terms and the lack of a consensus on definitions have several negative implications for research and clinical practice. The lack of a uniform, internationally recognized set of terms and definitions makes it difficult to compare results from different studies and to present evidence on CAM and its safe and effective application. Furthermore, the lack of consensus limits the transfer of research knowledge to health practitioners, impairing their ability to practice evidence-based medicine. The lack of appropriate terminology also renders the communication both within and among practitioners, researchers, educators, and most importantly patients, more complex and confusing. It is becoming clear, for example, that none of the terms are clearly understood by the general public [6]. This terminological confusion has real-world consequences: it prevents effective interprofessional collaboration between conventional and CAM practitioners, which may lead to the deterioration of patient-centered care.

In 2009–2010 the research team surveyed more than 200 leaders and experts in the field of CAM or IHC using a modified Delphi survey with the goal of: 1) investigating the appropriateness of the term CAM and its definition; and 2) exploring other possible term(s) to describe what is currently referred to as CAM [16]. The survey results suggested that although deficient, there are no alternatives for the terms CAM and IHC that can be used in both the scientific literature and public fora. The Delphi participants proposed two ways to circumvent these issues: since the CAM-related terms were found to be highly contextual (i.e. linked to research, practice, education, etc.), one needs 1) to identify the proper contexts of use in order to use the terms under appropriate circumstances; and 2) to revisit the current theoretical definitions of CAM and IHC to reflect and alleviate the concerns of academics and healthcare practitioners.

In order to explore the implications of these two propositions, we organized a two-phase consultation process in the form of a focus group of international leaders in the research and practice fields of CAM, IHC and conventional medicine, followed by an online forum, which included many more international practitioners and researchers. This paper reports on this two-phase consultation process, which aimed to clarify the contexts in which each of these terms can be used appropriately. The consensus-based definitions for both CAM and IHC are also presented.

## Methods

In November 2010, 13 international experts in the CAM field attended a one-day focus group to discuss the results of the Delphi survey on the appropriateness of the term CAM [16]. The participants were from different research and clinical backgrounds, representing both conventional medicine and CAM. These individuals had been chosen 1) based on their contributions to the healthcare literature with respect to CAM terminology, or 2) because they occupied an influential research or decision making position in the field. Participants represented four countries: Canada, the United States, Israel, and England.

The day’s tasks included: (1) clarifying the context within which CAM-related terms are most appropriately used, and (2) elaborating on a revised theoretical definition for both CAM and IHC, incorporating a list of key features of the definition suggested by the Delphi survey respondents. All sessions and tasks were guided by a moderator (independent from the research team). The focus group discussions were semi-structured: First, the results of the Delphi survey were presented and briefly discussed to familiarize the participants and clarify questions. Then, participants were given time to discuss the findings and their position until no new perspectives emerged. For example, contexts that emerged from the Delphi survey included: education, policy making, research and publication as well as communications among and between practitioners and patients. Each context was brought up along with relevant quotations from the survey. A two-dimensional table with contexts in the rows and terms in the columns was used to facilitate participation. The audio of each session was digitally recorded.

Definitions that emerged from the focus group were then discussed among an extended group of 28 international experts (the 13 focus group participants and 15 other individuals who could not attend the focus group due to scheduling or travelling difficulties) in a private online forum over a period of six weeks. Participants used aliases known only by the study’s investigators (to identify them in the forum). Proposed comments and modifications to the definitions were visible to all participants. Reminders were sent on a weekly basis to encourage participants to comment on the discussion threads. Audio recordings of the meeting as well as the forum discussion threads were analyzed using interpretive description. Prior to the focus group, research ethics approval for both inquiry steps was obtained from the University of Calgary’s Research Ethics Board. Informed written consent was obtained from each participant before the focus group. Participation in the forum discussion was considered implicit consent.

## Results

### Terms and their contexts

Similar to the Delphi survey respondents, the focus group participants quickly reached the consensus that different CAM-related terms have different purposes and that their use is highly contextual. As one participant put it, “*The purpose of a term is to identify something, and so terms may shift because of the focus of the discussion*.” (Focus Group [FG] participant 2) Thus, the focus group participants were given the task of clarifying terms according to context.

In light of the Delphi results, two umbrella terms were discussed (CAM and IHC), as well as the names of the specific disciplines subsumed under these labels. Even though participants generally agreed that we lack optimal definitions for these two umbrella terms and that all terms that have so far been introduced are to some extent problematic, CAM and IHC remained the most popular and accepted terms by far. The names of the specific disciplines were also deemed adequate in certain contexts. When debating whether CAM’s practice and research communities should come up with one or more terms to replace CAM, the general consensus was that a change in terminology would be a step backwards. As one focus group participant said: “*Language and power are closely intertwined. If we let go of the term CAM, it will be detrimental for the field.*” (FG participant 6) Another added: “[*In the USA,] if NCCAM lets go of CAM, the CAM disciplines, in particular, will be gone*. *It designates the CAM disciplines. It’s about research, it’s about enabling, it’s about professional development, it’s about boundaries, you know… it’s about inclusion. The fear is if you use IHC [over CAM], you will lose the focus. […] It’s an enfranchising term.*” (FG participant 7) On the other hand, a few participants remarked on a strong tendency for certain European governments to avoid using the term CAM. This might illustrate that, as one participant put it, “*the golden age of the CAM term is fading out*” in Europe.

The usefulness of umbrella terms was discussed at length. Most participants agreed that such terms, in particular CAM, were convenient especially, “*when you’re studying or researching a group of disciplines or therapies. […] We’re not going to identify each one of them. That’s the context in which we use the term CAM.*” (FG participant 5) Other participants provided examples from different settings. A participant concluded: “*We need a global term to refer to the field*.” (FG participant 7) Participants also acknowledged that the term CAM represents a “brand.” One commented: “*As the director of a graduate program, any of the other terms don’t help me, because nobody knows what we’re talking about whereas CAM, they do! And they say, oh yes, that’s all the other stuff.*” (FG participant 5) A consensus emerged on the benefits of using the term CAM after one participant questioned the group: “*By hanging together under an umbrella term, does that actually help the individual disciplines? Or is it better to seek independence by saying “I’ll take my chances, I’m an acupuncturist, I want to study acupuncture, I want to leave the CAM term and I’ll live by myself on an acupuncture term, and hope that funding agencies will fund me.” That’s the question? […] My feeling is at this stage, around the globe, there is more benefit to hanging together*.” (FG participant 5)

On the other hand, one participant cautioned that some practitioners and researchers have abused umbrella terms, using them as catchall terms to lump together any therapies or practitioners that do not qualify as biomedicine. For example, he explained that this strategy has provided those practitioners and researchers with ammunition to claim that CAM is ineffective. As one of the forum participants underscored, “*I do not think that meditation is the same as chiropractic or herbs or homeopathy. When things are all lumped, criticisms of one are lobbed against all, muddying the discussion.*” (Forum participant 14) Therefore, efforts should be made to define the scope of the research question and to refrain from using umbrella terms when another term that specifically refers to the discipline’s name could be used.

In parallel, participants were much in agreement that the use of umbrella terms should be restrained in the practitioner-patient dialogue for sake of clarity. If used, an umbrella term should be used only when referring to a group or practitioners or therapies, which is usually uncommon in the context of such a therapeutic relationship. One participant stressed the importance of referring to the discipline’s name by saying: “*What makes it alternative for practitioners and researchers [and thus justifies the use of an umbrella term] is the theory; for the patient, it’s the needles, the herbs, etc*.” (FG participant 8)

Of note, a majority of the focus group participants had major concerns about using the term “integrative medicine” as an alternative to “integrative healthcare,” and so the latter was employed for the discussion. Participants agreed that although “medicine” is a powerful word, its use is often limited to or associated with biomedicine. For this reason, participants believed that “healthcare” was a more inclusive term when used along with the term “integrative” and therefore was more adequate for the discussion. As one participant commented: “*There are issues around the terminology medicine. That’s one of the things that has come through the discussions in multiple disciplines, nursing being one of the forefront disciplines in the discussion, that medicine is limited to practitioners or professionals who have a medical license. So broadening the term to [the term] healthcare does make sense.”* (FG participant 3)

As alluded to in the Delphi survey, it became clear during the first session that the context of use should indicate which term to use. Focus group participants chose to add ‘philosophy’ to the original list of contexts compiled by the Delphi survey respondents. This context refers to the values and beliefs attached to the system within which practitioners and patients interact. Table 1 shows which terms are appropriate for which context, as determined by the participants. Participants had difficulty agreeing on which terms should be used in the contexts of practitioner-patient interactions and practitioner-practitioner interactions. Reference to the discipline’s specific name was favoured over the other two available options. Nevertheless, when an umbrella term is necessary, participants suggested the term CAM should be considered.

**Table 1:** Context of use of CAM-related terms

**Context/term**	**CAM**	**IHC**	**Discipline’s name**
Education of conventional and CAM practitioners			X
Policy making		X	
Philosophy		X	
Research (grant, publication)	X*		X
Practitioner-patient interaction	X*		X
Practitioner-practitioner interaction	X*		X

*When an umbrella term is necessary, i.e. when referring to a group of disciplines or providers.

### Definitions proposed

Overall, the participants agreed that despite their deficiencies, the terms CAM and IHC remain the best options. However, the definitions need to be revisited and clarified. In an effort to facilitate this process, the Delphi survey respondents were invited to enumerate features that should be included in new or revamped definitions of the terms CAM and IHC. These key concepts included: (1) therapeutic intent of the therapy or product (to distinguish, for example, between church going as a routine and praying specifically for a cure); (2) the use of purposefully identified regulated, licensed and well-established groups of practitioners and disciplines; (3) existence of a body of knowledge that suggests therapy might work; (4) patient-centeredness; (5) safety of the therapy or product when delivered and used as indicated; and (6) specific to IHC, a reference to the concept of collaborative work.

These concepts were presented to the focus group participants, who were then asked to assemble a new definition for CAM and IHC. Participants quickly devised a definition for IHC; the only point of uncertainty was whether the term CAM should be included in the definition. A slightly greater proportion of participants thought CAM should not be mentioned in the definition. Finding an acceptable definition for CAM, on the other hand, was more of a challenge. Despite vigorous discussion, the participants were unable to agree upon a comprehensive definition. Both definitions were subject to critiques and were subsequently modified over the course of the six weeks that the forum was open. The three authors performed interpretative analysis of the comments and made iterative modifications to the definitions. The resulting definitions are presented in Table 2.

**Table 2:** Suggested definitions


*CAM*: A broad range of therapeutic interventions developed and practiced by trained healthcare professionals and disciplines who have created bodies of knowledge that are used for education and training. These interventions are based on three important principles: (1) to treat the whole person; (2) to see the individual as a facilitator of health; (3) to see the body as having the inherent ability to heal itself.
*Integrative healthcare*: A system of healthcare that is patient-centered and collaborative, encompassing a diversity of therapeutic options [including CAM] that have been found to be safe, effective and informed by available evidence to achieve optimal health and healing.

## Discussion

Given the complexities of our healthcare system, finding appropriate definitions for terms like CAM is never easy. Nevertheless, it remains imperative that we do so, since increased clarity will promote knowledge translation and improve communication, especially with regard to research. Accordingly, the most effective way to improve the definition of CAM is to determine the proper contexts in which it should be used. This paper demonstrates that (1) CAM and other terms can be used more effectively when the proper context is identified; and (2) although revised definitions of terms related to CAM are necessary, international consensus on the matter remains elusive.

This research project also suggests that the field of CAM itself may be undergoing a transition in both practice and research. A large majority of the Delphi survey respondents would like research and healthcare practice communities to revisit the definitions of both CAM and IHC. Nevertheless, many participants conceded the futility of the exercise at this point in time, claiming that doing so would only result in more confusion and arguments. As foreseen by Ernst and colleagues in 1995, as there is a shift to diversify the frameworks of medicine, [7] the practitioner and researcher communities are at a crossroads. Should we move beyond the term CAM? It seems likely that the introduction of the term IHC in the 1990’s represents a first step – at least ideologically – towards CAM’s integration into mainstream healthcare. This integration process might eventually evolve into the purest meaning of an IHC system: a diversity of therapeutic options offered throughout the healthcare system, with no particular differentiation between any healthcare paradigms. As suggested by the participants, a long term outcome of this transition may be that biomedicine will eventually co-opt CAM fields. Drawbacks of this scenario may include, but are not limited to: a more curative rather than preventive approach to healthcare, less empowerment for the patient, and the overall devaluation of non-biomedical services and knowledge [17-19]. Nevertheless, it appears CAM is beginning to be brought into a relationship with biomedicine, signalling a long and challenging process of integration for contemporary healthcare systems.

One indication that a transition is taking place is the finding that practitioners and educators should limit the use of the terms CAM and IHC when referring to CAM-related treatments with their patients and students, for the sake of clarity. Tataryn and Verhoef’s “integration pyramid” showed that patients/consumers are ideally positioned to exert bottom-up pressure on the entire healthcare system, including at the practitioner, clinical, institutional, and policy making levels [20]. The demand that the public and healthcare practitioners resist using CAM-related umbrella terms indicates that we are slowly moving beyond the CAM era. This may create a ripple effect and push the transition further, considering that changes and transformations at the patient level have a powerful influence over higher levels of the integration pyramid [20].

Another indication of this transition of CAM, as aspects are being subsumed into biomedical practice, is the definition for the label CAM generated by the study participants. The three principles of the definition, in fact, could readily be applied to conventional treatments, while CAM might not always necessarily be delivered according to those principles (e.g. the administration of an herbal therapy or a manipulative adjustment could be sometimes provided in a quite reductionist fashion). Thus the boundaries between conventional medicine and CAM are being challenged. This being said, in CAM these principles are assumed to be interrelated, and in the practice of conventional medicine, these principles are less explicit and less common, especially the third principle about vitalism.

Our findings suggest that the philosophy related to the CAM field and its terms will eventually evolve. Initiatives that aim to clarify the terms of interest, such as this present project or the working definition of Wieland and colleagues, will support this transition. The term CAM remains in use simply because of its popularity and because there is no acceptable alternative. During this research project, allusion was often made to a love-hate relationship with the term: with all its faults, at least the term CAM offers branding at political, and to a certain extent, public levels that no other terms have succeeded in obtaining [6,21,22]. It is important to be mindful of the fact that adequate terminology is required at all times, even while the transition takes place.

The transition towards another term (or terminology) will happen, but not before certain issues are addressed. First, the fields of healthcare research and practice need to acknowledge that CAM disciplines are parts of a whole. Then, we need a clear understanding of each of the parts that are coming together, from the perspective of CAM, biomedical and any other healthcare systems involved. Indeed, there are dangers associated with distancing the current CAM-related healthcare terminology too quickly from its complementary and alternative meaning. As mentioned above, the main danger involves the adoption of CAM by the biomedical system. While the field of CAM has developed extensively over the last decades, some aspects of such an ultimately complex IHC system are still quite fragile, especially from a policy-making point of view. For example, there may be ambiguity with regards to establishing leadership roles and responsibilities when CAM and conventional medical practitioners collaborate within an IHC system, from a clinical and patient perspective.

### Study limitations

One of the limitations of our study is that representatives of the general public (CAM users or otherwise) were not included. Also, despite efforts to recruit European experts and practitioners, their voices may not have been represented as well as those of North Americans. Thus, caution must be taken when interpreting the results from an international perspective.

## Conclusions

Change occurs when principal stakeholders express the need for it and are given the freedom and encouragement to attempt it [23,24] The participants of this research project have clearly indicated the need for the wheels to be set in motion but recognise that the term CAM will still be used while the research and practice communities move towards another healthcare paradigm. The general public should be included in the following steps of the process as critical stakeholders of this paradigm change.

## Abbreviations

CAM, Complementary and alternative medicine; FG, Focus group; IHC, Integrative healthcare; NCCAM, National Center for Complementary and Alternative Medicine; US, United States of America.

## Competing interests

The authors declare that they have no competing interests.

## Authors’ contributions

All authors participated in the conception and design of the study. IG carried out the analyses and drafted the manuscript. KTA and MV helped with the interpretation of the data. All authors read and approved the final manuscript.

## Pre-publication history

The pre-publication history for this paper can be accessed here:

http://www.biomedcentral.com/1472-6882/12/131/prepub
